# Downregulation of Carbonic Anhydrase IX Promotes *Col10a1* Expression in Chondrocytes

**DOI:** 10.1371/journal.pone.0056984

**Published:** 2013-02-18

**Authors:** Toshifumi Maruyama, Yoichi Miyamoto, Gou Yamamoto, Atsushi Yamada, Kentaro Yoshimura, Tetsuo Suzawa, Masamichi Takami, Tomohito Akiyama, Marie Hoshino, Fuminori Iwasa, Noriharu Ikumi, Tetsuhiko Tachikawa, Kenji Mishima, Kazuyoshi Baba, Ryutaro Kamijo

**Affiliations:** 1 Department of Biochemistry, School of Dentistry, Showa University, Shinagawa, Tokyo, Japan; 2 Department of Prosthodontics, School of Dentistry, Showa University, Shinagawa, Tokyo, Japan; 3 Division of Pathology, Department of Oral Diagnostic Sciences, School of Dentistry, Showa University, Shinagawa, Tokyo, Japan; University of Maryland School of Medicine, United States of America

## Abstract

Carbonic anhydrase (CA) IX is a transmembrane isozyme of CAs that catalyzes reversible hydration of CO_2_. While it is known that CA IX is distributed in human embryonic chondrocytes, its role in chondrocyte differentiation has not been reported. In the present study, we found that *Car9* mRNA and CA IX were expressed in proliferating but not hypertrophic chondrocytes. Next, we examined the role of CA IX in the expression of marker genes of chondrocyte differentiation *in vitro*. Introduction of *Car9* siRNA to mouse primary chondrocytes obtained from costal cartilage induced the mRNA expressions of *Col10a1*, the gene for type X collagen α-1 chain, and *Epas1*, the gene for hypoxia-responsible factor-2α (HIF-2α), both of which are known to be characteristically expressed in hypertrophic chondrocytes. On the other hand, forced expression of CA IX had no effect of the proliferation of chondrocytes or the transcription of *Col10a1* and *Epas1*, while the transcription of *Col2a1* and *Acan* were up-regulated. Although HIF-2α has been reported to be a potent activator of *Col10a1* transcription, *Epas1* siRNA did not suppress *Car9* siRNA-induced increment in *Col10a1* expression, indicating that down-regulation of CA IX induces the expression of *Col10a1* in chondrocytes in a HIF-2α-independent manner. On the other hand, cellular cAMP content was lowered by *Car9* siRNA. Furthermore, the expression of *Col10a1* mRNA after *Car9* silencing was augmented by an inhibitor of protein kinase A, and suppressed by an inhibitor for phosphodiesterase as well as a brominated analog of cAMP. While these results suggest a possible involvement of cAMP-dependent pathway, at least in part, in induction of *Col10a1* expression by down-regulation of *Car9*, more detailed study is required to clarify the role of CA IX in regulation of *Col10a1* expression in chondrocytes.

## Introduction

Carbonic anhydrases (CAs, EC 4.2.1.1) are zinc metalloenzymes that catalyze reversible hydration-dehydration of carbon dioxide and bicarbonate (CO_2_ + H_2_O ↔ HCO_3_^-^ + H^+^). In higher vertebrates, at least 13 active CA isozymes have been identified, namely CAs I, II, III, IV, VA, VB, VI, VII, IX, XII, XIII, XIV, and XV. They are grouped by their subcellular localization, as CAs I, II, III, VII, and XIII reside in cytosol, VA and VB are found in mitochondria, VI is secreted in saliva and milk, and IV, IX, XII, XIV, and XV are anchored in the plasma membrane. In addition, 3 acatalytic CA-related proteins (CARPs) have been reported, namely cytosolic CARP VIII, and extracellular CARPs X and XI [1]. Among these CA isozymes, CA IX has been shown to be overexpressed in solid tumors, and has roles in tumor growth promotion, metastasis, and poor responsiveness to radio- and chemotherapy [2], whereas its expression in normal tissues is generally quite low, except for some locations including gastric mucosa [3]. Inhibition of CA IX in tumor cells by chemicals or specific antibodies is regarded as a promising strategy for cancer treatment [4]. CA IX, with a molecular weight of 54/58 kDa, consists of an N-terminal proteoglycan-like domain, CA catalytic domain, transmembrane domain, and short cytoplasmic tail at the C-terminus [5], and forms a homodimer by a disulfide bridge between the catalytic domains [6]. It is known that *CAR9*, the gene coding for CA IX, is one of the most hypoxia-sensitive genes with a hypoxia-responsive element in its promoter region [7], while hypoxia inducible factor (HIF)-1α but not HIF-2α activates *CAR9* transcription [8]. The hypoxic environment of solid tumors is believed to be an important factor to induce CA IX overexpression.

In endochondral bone formation, mesenchymal cells migrate to sites of future skeletogenesis and differentiate into chondrocytes. Chondrocytes proliferate and sequentially differentiate from resting chondrocytes into proliferating chondrocytes, and then into hypertrophic chondrocytes, which finally induce vasculature, then mineralize and undergo apoptosis. Cartilage is one of the most avascular tissues and its development is promoted under hypoxic conditions [9], while HIF-1α is distributed in the interior region of growth plates where highly hypoxic chondrocytes are located [10]. It was also reported that HIF-1α plays important roles in the survival of chondrocytes as well as matrix production by chondrocytes [11]. HIF-1α directly promotes the expression of *Sox-9*, a master gene of chondrogenesis, by binding to its promoter region [12], thus it is required for normal development of cartilage [13]. In addition, it was recently reported that hypertrophic chondrocytes express mRNA for *Epas1*, the gene coding a transcription factor HIF-2α. HIF-2α is known to promote the expression of type X collagen α-1 chain (*Col10a1*), a marker gene for hypertrophic chondrocytes, and those for terminally differentiated chondrocytes, such as matrix metalloproteinase-13 (*Mmp13*), and vascular endothelial growth factor A (*Vegfa*) [14].

We recently found that CA II, an isozyme of CA IX distributed in cytosol, regulates the differentiation of ameloblasts by modulating cytosolic pH [15]. Although immunohistochemical analysis revealed that CA IX is distributed in chondrocytes in human embryos [16], to the best of our knowledge, there is no report of the function of CA IX in chondrocytes. Considering that CA IX expression is induced by HIF-1α and CA IX is involved in tumor growth, it is considered important to investigate the possible involvement of CA IX in the growth, differentiation, and function of chondrocytes. In this study, we explored the expression of CA IX in epiphyseal cartilage in mice and its role in the expression of differentiation-related genes in chondrocytes *in vitro*.

## Results

### CA IX Was Distributed in Stationary and Proliferating Chondrocytes, but not in Hypertrophic Chondrocytes in Mouse Epiphyseal Cartilage

**Figure 1:**
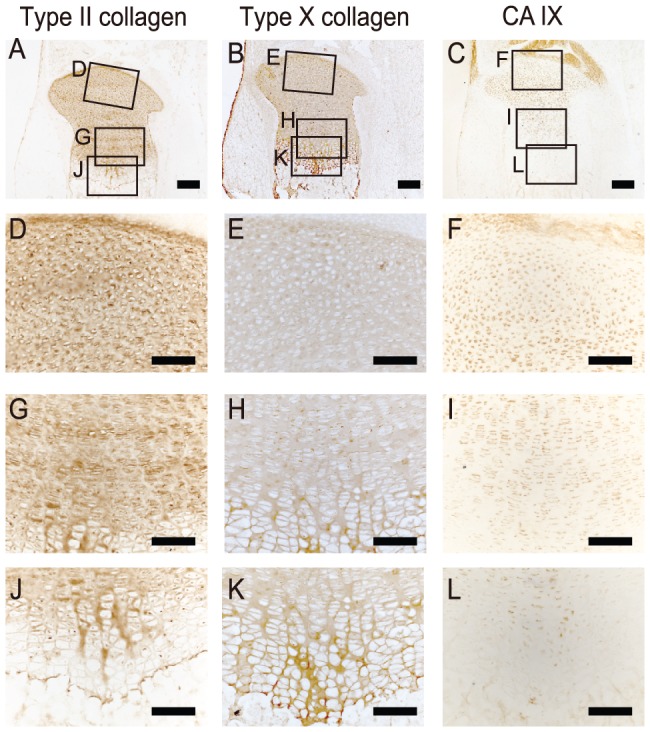
Localization of CA IX in epiphyseal cartilage. Frozen sections of lower limbs excised from 1-day-old postnatal ddY mice were analyzed for immunolocalization of type II collagen (**A**, **D**, **G**, **J**), type X collagen (**B**, **E**, **H**, **K**), and CA IX (**C**, **F**, **I**, **L**). Photographs show the entire epiphysis with lower magnification (**A**–**C**), regions containing stationary to round-shaped proliferating chondrocytes (**D**–**F**), columnar chondrocytes proliferating to pre-hypertrophic chondrocytes (**G**–**I**), and hypertrophic chondrocytes (**J**–**L**) at higher magnifications. The regions magnified (**D**–**L**) were shown in the photographs of the entire epiphysis (**A**–**C**). Bars are 0.2 mm (A–C) and 0.1 mm (D–L).

It has been reported that CA IX is distributed in chondrocytes in human embryos [16]. However, the role of this enzyme in differentiation and metabolism of chondrocytes has not been investigated. To gain insight into the function of CA IX in differentiation of chondrocytes in the growth plate, we first examined its immunohistochemical localization in epiphyseal cartilage from neonatal mouse tibia, and compared it with that of type II and type X collagens, marker proteins of chondrocytes and hypertrophic chondrocytes, respectively (Figure 1). Type II collagen was distributed throughout the cartilage (Figures 1A, D, G, and J), while type X collagen was localized in hypertrophic regions (Figures 1B, E, H, and K). On the other hand, CA IX immunoreactivity was detected in round and columnar proliferating chondrocytes, especially those in the central portion of the growth plate (Figure 1C). The protein expression of CA IX gradually decreased along with the progression of chondrocyte differentiation (Figures 1C, F, and I) and was scarcely detected in hypertrophic chondrocytes (Figure 1L), which was in clear contrast to type X collagen immunoreactivity (Figures 1B, E, H, and K).

**Figure 2:**
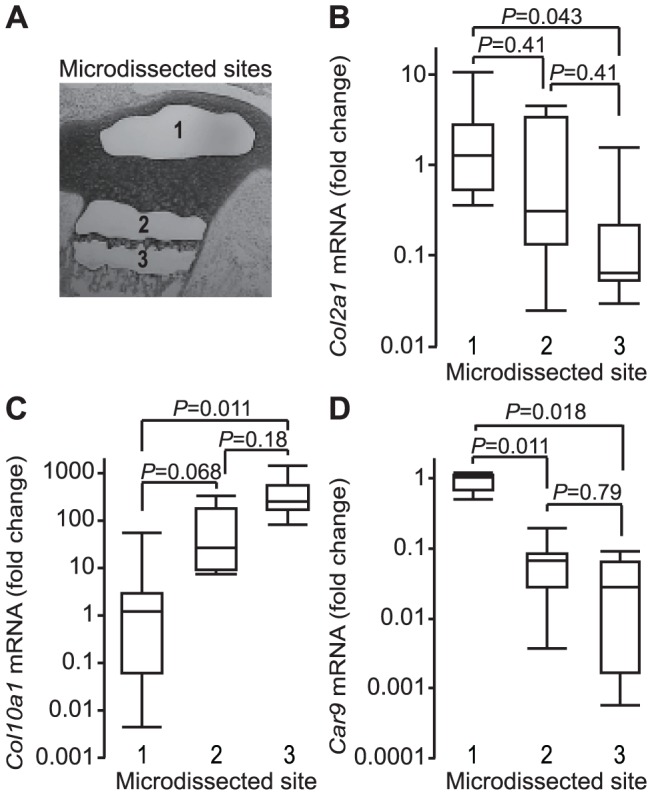
Expression of *Car9* mRNA in epiphyseal cartilage. Frozen sections of lower limbs excised from 1-day-old postnatal ddY mice were laser microdissected (**A**) and analyzed for the mRNA expressions of *Col2a1* (**B**), *Col10a1* (**C**), and *Car9* (**D**). **A.** Regions consisting of round-shaped proliferating chondrocytes (**1**), columnar proliferating and pre-hypertrophic chondrocytes (**2**), and hypertrophic chondrocytes (**3**) were isolated from epiphyseal cartilage sections sliced by laser microdissection. **B.** Expression of *Col2a1* mRNA in the microdissected specimens (**1**, **2**, **3**). **C.** Expression of *Col10a1* mRNA in the microdissected specimens (**1**, **2**, **3**). **D.** Expression of *Car9* mRNA in the microdissected specimens (**1**, **2**, **3**). The expression of *Col2a1*, *Col10a1*, and *Car9* were quantitatively analyzed by real-time RT-PCR. The expression level of each gene was normalized to that of *Gapdh* and expressed as a value relative to that obtained in region **1**. Data are expressed by boxplots (the sample maximum, the upper quartile, the median, the lower quartile, and the minimum observation). *P*-values obtained by Steel-Dwass test were indicated (n = 6).

To ascertain differentiation-dependent changes in CA IX distribution, we quantitatively analyzed the expressions of *Col2a1*, *Col10a1*, and *Car9* mRNA in regions that contained round proliferating chondrocytes, columnar proliferating chondrocytes, and hypertrophic chondrocytes in slices of epiphyseal cartilage prepared by laser microdissection (Figure 2A). While the expression of *Col2a1* mRNA showed a tendency to decrease with progression of differentiation (Figure 2B). The expression of *Col10a1* mRNA was prominent in hypertrophic region (Figure 2C). On the other hand, the highest level of *Car9* mRNA expression was detected in regions containing round proliferating chondrocytes, while that was decreased in a differentiation-dependent manner and became very low in hypertrophic regions, where *Col10a1* mRNA was highly expressed (Figure 2D). These results clearly indicate that the expression of *Car9* mRNA is dependent on the differentiation stage of chondrocytes and down-regulated in hypertrophic chondrocytes. It was previously reported that transcription of the *Car9* gene is promoted by HIF-1α [8], a transcription factor known to reside in chondrocytes in the growth plate proliferating zone at early differentiation stages [10], [14]. Therefore, it is conceivable that the expression of CA IX protein in epiphyseal cartilage is regulated by HIF-1α at the transcriptional level.

### *Car9* siRNA Induced Increased Expression of *Col10a1* mRNA in Primary Chondrocytes

**Figure 3:**
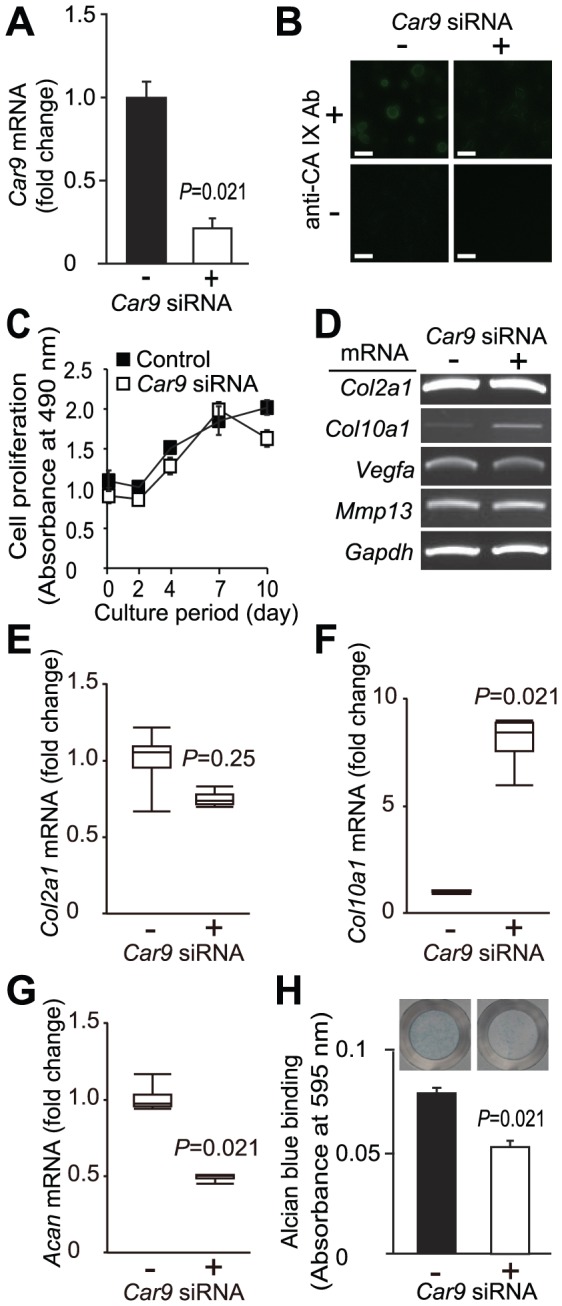
Effects of *Car9* siRNA on chondrocyte proliferation and expressions of marker genes of chondrocyte differentiation. *Car9* siRNA or control siRNA was introduced into primary chondrocytes isolated from the rib cages of 1-day-old postnatal ddY mice. **A.** The expression of *Car9* mRNA was analyzed by real-time RT-PCR at 48 hours after introduction of *Car9* siRNA or control siRNA, with the level normalized to that of *Gapdh*. Data are expressed as the mean ± SD (n = 4) for fold changes caused by introduction of *Car9* siRNA. *P*-value obtained by two-tailed Mann-Whitney *U*-test (n = 4) indicated that the *Car9* siRNA used in this study significantly lowered the expression level of *Car9* mRNA (α = 0.05). **B.** Expression of CA IX protein in chondrocytes was detected by immunocytochemical staining at 48 hours after introduction of control siRNA (left 2 panels) or *Car9* siRNA (right 2 panels). Lower 2 panels show the results obtained without primary antibody against CA IX. Bar, 50 µm. **C.** *Car9* siRNA (unfilled square) or control siRNA (filled square) was introduced to chondrocytes on day 0. Proliferation of chondrocytes was assessed spectrophotometrically using CellTiter 96^®^ Aqueous One Solution. Data are expressed as the mean ± SD (n = 4). At each time point, Mann-Whitney *U*-test with Bonferroni correction was performed to evaluate the difference between control siRNA- and *Car9* siRNA-introduced cells. No significant difference between the values was indicated at any time point (α = 0.01). **D.** At 48 hours after introduction of siRNAs, expressions of mRNAs for *Car9*, *Col2a1*, *Col10a1*, *Vegfa*, and *Mmp13* were analyzed by RT-PCR. **E-G.** The expressions of *Col2a1* (**E**), *Acan* (**F**), and *Col10a1* (**G**) were quantitatively analyzed by real-time RT-PCR. The expression level of each gene was normalized to that of *Gapdh*. Data are expressed by boxplots (n = 4) in the fold change by introduction of *Car9* siRNA (the sample maximum, the upper quartile, the median, the lower quartile, and the minimum observation). *P*-values determined by two-tailed Mann-Whitney U-test are indicated. **H.** At 4 days after introduction of *Car9* or control siRNA, chondrocyte cultures were stained with Alcian blue. Alcian blue bound to the cell matrix was extracted and determined spectrophotometrically (n = 4). A *P*-value determined by two-tailed Mann-Whitney *U*-test is indicated. Typical photographs are shown above the columns.

Our finding of differentiation stage-dependent expression of CA IX in growth plate specimens (Figures 1 and 2) raised the possibility that this enzyme is involved in chondrocyte differentiation. To explore that possibility, we examined the effects of *Car9* siRNA on the expression of several genes in primary chondrocytes, including marker genes of chondrocyte differentiation. As shown in Figure 3A, the *Car9* siRNA used in this study significantly suppressed the expression of *Car9* mRNA (α = 0.05). In addition, fluorescent immunostaining for CA IX showed a lowered expression of CA IX protein after introduction of *Car9* siRNA (Figure 3B). Proliferation of chondrocytes was not suppressed significantly by introduction of *Car9* siRNA (Figure 3C, α = 0.01). Next, we examined the expressions of marker genes related to differentiation of chondrocytes, including *Col2a1*, *Col10a1*, *Vegfa*, and *Mmp13*, using reverse transcription (RT)-polymerase chain reaction (PCR) (Figure 3D). Expression of *Col2a1*, expressed by chondrocytes irrespective of their differentiation stage, was not changed by *Car9* silencing. The *Col10a1* gene is known to be expressed in hypertrophic chondrocytes, and we found that its mRNA expression was up-regulated by introduction of *Car9* siRNA. On the other hand, the expressions of *Vegfa* and *Mmp13*, both of which are specifically observed in terminally differentiated hypertrophic chondrocytes in growth plates, were not affected by *Car9* siRNA. To ascertain the augmented expression of *Col10a1* in *Car9*-silenced chondrocytes, the expression level of *Col10a1* mRNA was quantitatively analyzed using real-time RT-PCR along with those of *Col2a1* and aggrecan (*Acan*) (Figures 3E–G). *Car9* siRNA caused an 8-fold increase in *Col10a1* expression (Figure 3F). *Acan* as well as *Col2a1* are known to be expressed by both proliferating and hypertrophic chondrocytes. While the mRNA level of *Col2a1* was not affected (Figure 3E), that of *Acan* was suppressed to one-half by introduction of *Car9* siRNA (Figure 3G). In parallel with the lowered expression of *Acan* mRNA, introduction of *Car9* siRNA induced a 29.6% decrease in the amount of Alcian blue bound to the chondrocyte culture at 4 days after the siRNA introduction (Figure 3H), indicating that the amounts of proteoglycans including aggrecan were decreased by *Car9* silencing.

### *Epas1* Expression Was Up-regulated by *Car9* siRNA in Primary Chondrocytes

**Figure 4:**
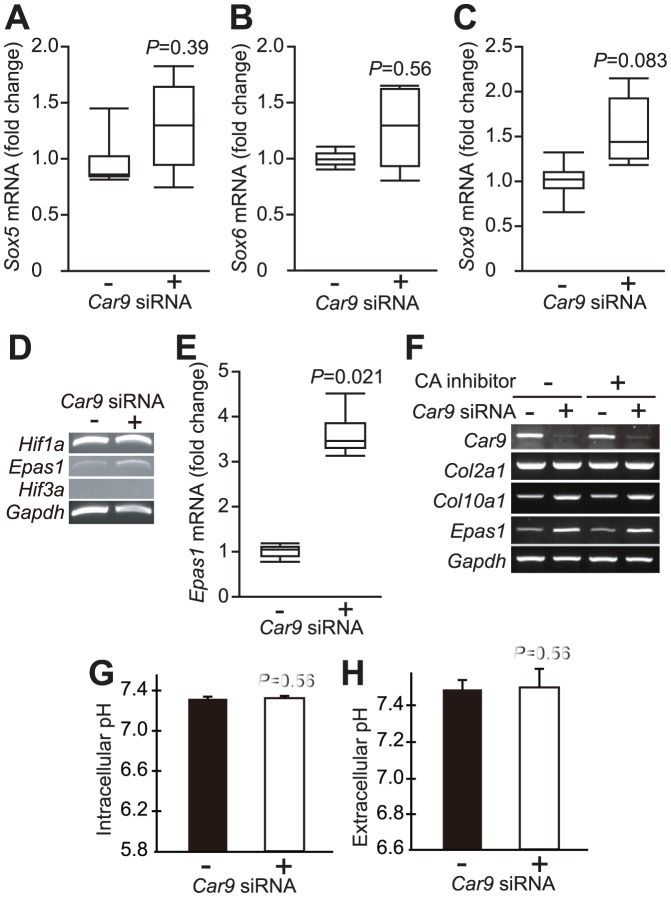
Effects of *Car9* siRNA on expressions of transcription factors related to chondrocyte differentiation. *Car9* siRNA (+) or control siRNA (-) was introduced into mouse primary chondrocytes. **A-C.** The expressions of *Sox5* (**A**), *Sox6* (**B**), and *Sox9* (**C**) were quantitatively analyzed by real-time RT-PCR at 48 hours after introduction of the siRNAs. The expression level of each gene was normalized to that of *Gapdh*. Data are expressed by boxplots (n = 4) for fold changes caused by introduction of *Car9* siRNA (the sample maximum, the upper quartile, the median, the lower quartile, and the minimum observation). **D**. The expressions of mRNAs for *Hif1a*, *Epas1*, *Hif3a*, and *Gapdh* were analyzed by RT-PCR at 48 hours after introduction of *Car9* siRNA or control siRNA. **E**. The expression of *Epas1* was quantitatively analyzed by real-time RT-PCR at 48 hours after introduction of the siRNAs. Data are expressed by boxplots **F**. Twenty-four hours after introduction of *Car9* siRNA or control siRNA, primary chondrocytes were additionally cultured for 24 hours in the presence or absence of 50 µM 4-(2-aminoethyl)-benzenesulfonamide, an inhibitor of extracellular CAs. Then, the expressions of mRNAs for *Car9*, *Col2a1*, *Col10a1*, *Epas1*, and *Gapdh* were analyzed by RT-PCR. **G** and **H**. Intracellular (**G**) and extracellular (**H**) pH values were determined in primary chondrocytes at 48 hours after introduction of *Car9* siRNA and control siRNA. Data are expressed as the mean ± SD of 4 experiments. *P*-values determined by two-tailed Mann-Whitney U-test are indicated.

Although *Sox5*, *Sox6*, and *Sox9* are genes for well-known transcription factors that control chondrocyte differentiation, their expressions were not shown to be influenced by *Car9* siRNA (Figures 4A–C). It was recently reported that HIF-2α, a transcription factor encoded by *Epas1*, is expressed in terminally differentiated growth plate chondrocytes [17] and activates *Col10a1* gene [14]. We examined the effects of introduction of *Car9* siRNA on the mRNA expressions of *Epas1* and other hypoxia-related transcription factors, namely *Hif1a* and *Hif3a*, using RT-PCR (Figure 4D). While the expression of *Hif1a* mRNA in primary chondrocytes was not affected, that of *Epas1* was up-regulated by introduction of *Car9* siRNA. *Hif3a* mRNA was not detected in chondrocytes regardless of the level of *Car9* expression. Real-time RT-PCR analysis revealed that *Car9* siRNA had little influence on the mRNA expressions of *Sox5*, *Sox6*, and *Sox9* (Figures 4A–C), whereas it induced a 3-fold increase in the mRNA level of *Epas1* (Figure 4E).

### CA IX Activity Was not Involved in Regulation of *Col10a1* and *Epas1* Expressions

We previously reported that CA II, the most abundant CA in cytosol, plays a pivotal role in differentiation of ameloblasts via intracellular pH-dependent regulation of c-Jun N-terminal kinase (JNK) activity [15]. To clarify that the decreased enzymatic activity of CA IX is involved in up-regulated expressions of mRNAs for *Col10a1* and *Epas1* in *Car9*-silenced chondrocytes, cells were cultured in the presence or absence of an inhibitor for CA IX, 4-(2-aminoethyl)-benzenesulfonamide, after introduction of *Car9* or control siRNA. As shown in Figure 4F, the CA IX inhibitor did not affect the mRNA expression of either *Col10a1* or *Epas1*. In addition, we determined intracellular and extracellular pH values in primary chondrocytes after introduction of *Car9* or control siRNA. Despite the fact that CA IX is one of the enzymes that regulates cellular pH, *Car9* siRNA showed little effect on intra- or extracellular pH in chondrocytes (Figures 4G and H). These results indicate that the catalytic activity of CA IX is dispensable for regulation of expression of these genes.

### *Col10a1* Expression Was Up-regulated by *Car9* siRNA in Primary Chondrocytes Cultured in A Hypoxic Condition

**Figure 5:**
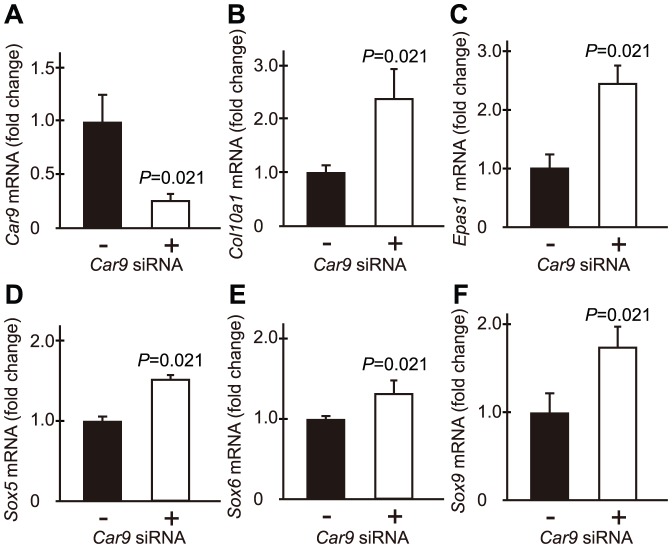
Effects of *Car9* siRNA on the expressions of transcription factors related to chondrocyte differentiation in primary chondrocytes cultured under a hypoxic condition. *Car9* siRNA (+) or control siRNA (-) was introduced into mouse primary chondrocytes under a hypoxic condition (5% CO_2_ and 95% N_2_). The cells were additionally cultured for 48 hours under the same condition. The expressions of *Car9*, *Col10a1*, *Epas1*, *Sox5*, *Sox6*, and *Sox9* were quantitatively analyzed by real-time RT-PCR. The expression level of each gene was normalized to that of *Gapdh*. Data are expressed as the mean ± SD (n = 4) in the fold change by introduction of *Car9* siRNA. *P*-values determined by two-tailed Mann-Whitney U-test are indicated.

In a separate experiment, we examined the effect of *Car9* silencing on mRNA expressions of *Car9*, *Col10a1*, *Sox5*, *Sox6*, *Sox9*, and *Epas1* in chondrocytes cultured under a hypoxic condition (Figure 5). There was a scant difference between the increments in *Col10a1* expression in chondrocytes under a hypoxic condition as compared to chondrocytes under normoxia. As with *Col10a1* expression, oxygen tension did not affect the level of *Epas1* induction by introduction of *Car9* siRNA. On the other hand, the expression levels of *Sox5*, *Sox6*, and *Sox9* were increased in *Car9*-silenced chondrocytes cultured under a hypoxic condition.

### Forced Expression of CA IX did not Affect Expression of *Col10a1* or *Epas1* in chondrocytes

**Figure 6:**
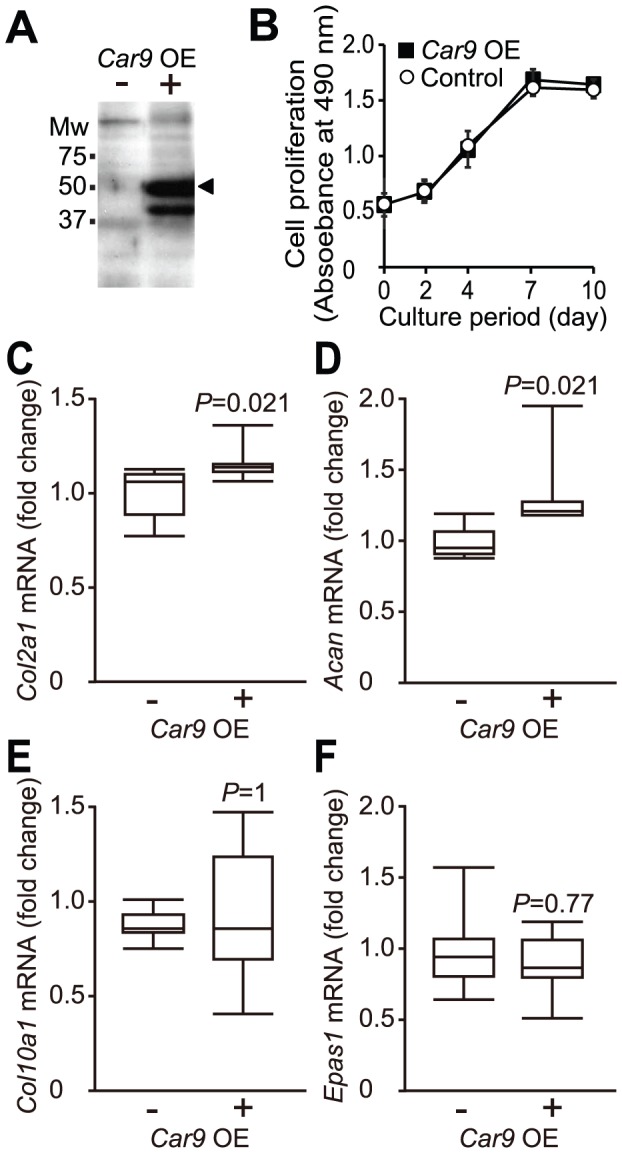
Effects of forced expression of CA IX on chondrocyte proliferation and mRNA expression of *Col2a1*, *Acan*, *Col10a1*, and *Epas1*. Primary mouse chondrocytes were transfected with a CA IX-expression plasmid (+) or its control plasmid (-). A. At 48 hours after transfection, the expression of CA IX (58kDa) was assessed by western blot analysis using anti-mouse CA IX antibody. CA IX band is indicated by an arrowhead. B. Proliferation of chondrocytes introduced with CA IX-expression (filled square) and control (unfilled circle) plasmids was assessed spectrophotometrically using CellTiter 96^®^ Aqueous One Solution. Data are expressed as the mean ± SD (n = 4). At each time point, Mann-Whitney *U*-test with Bonferroni correction was performed to evaluate the difference between control plasmid- and CAIX-expression plasmid-transfected cells. No significant difference between the values was indicated at any time point (α = 0.01). C-F. The expressions of *Col2a1* (C), *Acan* (D), *Col10a1* (E), and *Epas1* (F) were quantitatively analyzed by real-time RT-PCR, with the expression level of each normalized to that of *Gapdh*. Data are expressed by boxplots (n = 6) for fold changes caused by introduction of *Car9* siRNA (the sample maximum, the upper quartile, the median, the lower quartile, and the minimum observation). *P*-values determined by two-tailed Mann-Whitney *U*-test are indicated.

To examine whether overexpression of CA IX suppresses the expression of *Col10a1* and *Epas1* mRNAs in chondrocytes contrary to the reactions after introduction of *Car9* siRNA (Figures 3 and 4), we introduced an expression plasmid of CA IX or its empty plasmid into mouse primary chondrocytes. Two major bands were detected in cells introduced with the CA IX expression plasmid by Western blot analysis using an antibody against mouse CA IX (Figure 5A).The molecular weight of mouse CA IX (58kDa) indicates that the upper band is that for CA IX. It is known that CA IX promotes tumor cell growth [2]. However, overexpression of CA IX did not affect the proliferation of chondrocytes (Figure 6B). The expression of mRNAs for *Col2a1* and *Acan* was upregulated by introduction of the expression plasmid of CA IX (Figures 6C and D). On the other hand, that of mRNAs for *Col10a1* or *Epas1* was not changed by forced expression of CA IX (Figures 6E and F).

### *Epas1* and *Col10a1* Expressions Were Independently Regulated by CA IX

**Figure 7:**
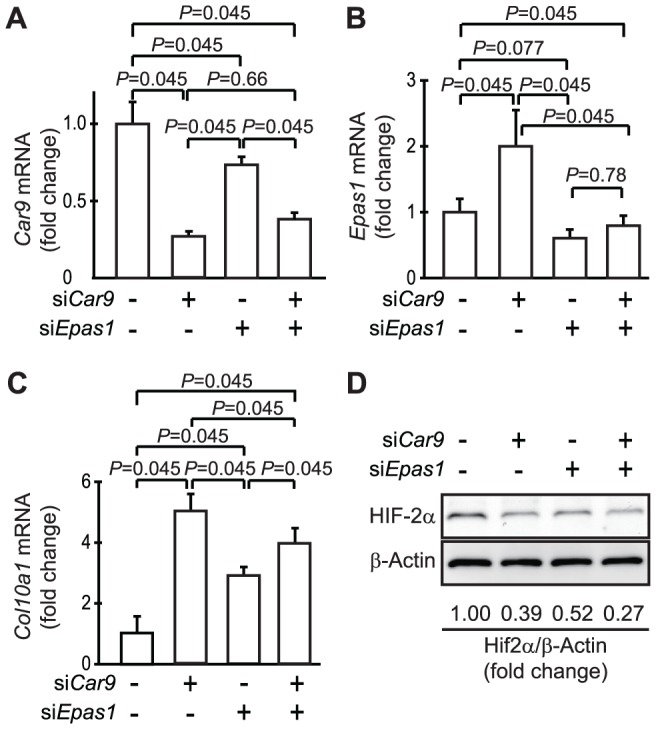
Independent regulation of expressions of *Epas1* and *Col10a1* by CA IX. *Car9* siRNA (si*Car9*) and *Epas1* siRNA (si*Epas1*) were simultaneously introduced into primary chondrocytes. The expression levels of mRNAs for *Car9* (**A**), *Epas1* (**B**), and *Col10a1* (**C**) were quantitatively analyzed by real-time RT-PCR. The expression level of each gene was normalized to that of *Gapdh* and is shown as the relative value to that obtained with cells introduced with only control siRNA (leftmost column in each graph). Data are expressed as the mean ± SD of 5 experiments. *P*-values obtained by Steel-Dwass test were indicated. **D.** The expression of HIF-2α protein in chondrocytes was examined by western blot analysis at 48 hours after introduction of siRNAs for *Car9* (si*Car9*) and/or *Epas1* (si*Epas1*). The expression level of HIF-2α in each cellular extract was normalized by that of β-actin. The values under the photographs show the fold change in HIF-2α/β-actin ratio by the introduction of siRNAs for *Car9* and *Epas1* relative to that obtained by the introduction of control siRNA.

The results described above suggested possible involvement of increased expression of *Epas1* in induction of *Col10a1* expression following down-regulation of CA IX in chondrocytes. Thus, we examined the effects of simultaneous silencing of *Epas1* and *Car9* on the expressions of *Car9* (Figure 7A), *Epas1* (Figure 7B), and *Col10a1* (Figure 7C) to elucidate the relationship between these 2 up-regulated genes. Contrary to our expectation, *Epas1* siRNA up-regulated the expression of *Col10a1* mRNA (Figure 7C), while it also slightly but significantly suppressed the expression of *Car9* mRNA (Figure 7A, α = 0.05). In addition, *Car9* siRNA augmented the expression of *Col10a1* even in chondrocytes co-introduced with *Epas1* siRNA (Figure 7C, α = 0.05). These results indicate that, if any interactions exist, the up-regulations of *Epas1* and *Col10a1* in *Car9*-silenced chondrocytes are independent events. Western blot analysis of the expression of HIF-2α protein in chondrocytes revealed that not only *Epas1* siRNA but also *Car9* siRNA lowered the expression level of HIF-2α (Figure 7D), despite the increased expression of *Epas1* mRNA in *Car9* siRNA-introduced cells (Figure 7B). These results indicate that HIF-2α did not play an important role in the induction of *Col10a1* expression in the *Car9*-silenced chondrocytes.

### Protein Kinase A Pathway Was Possibly Involved in *Car9*-siRNA-induced Up-regulation of *Col10a1* Expression

**Figure 8:**
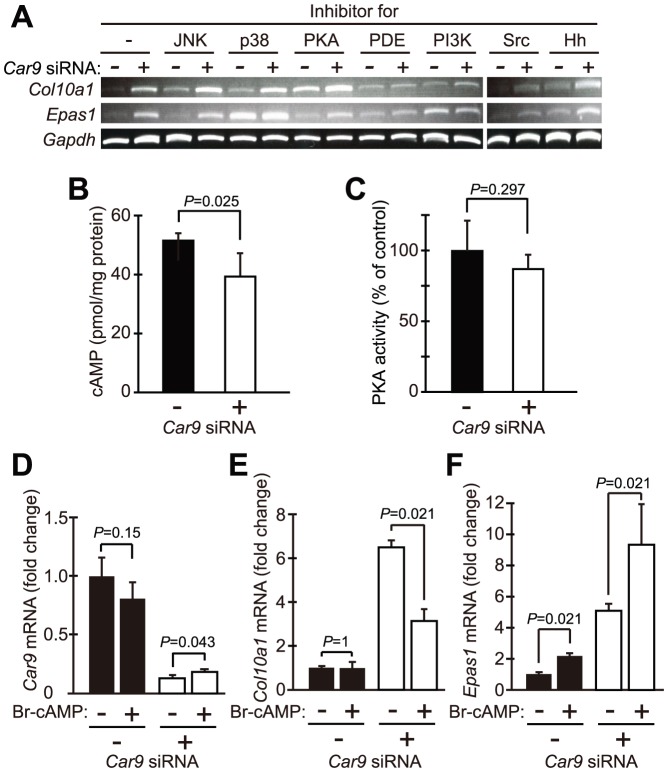
Intracellular signaling cascades involved in regulation of *Col10a1* expressions by CA IX. *Car9* siRNA or control siRNA was introduced into primary chondrocytes. **A.** At 24 hours after introduction of the siRNAs, the culture medium was changed to that containing the JNK inhibitor SP600125 (30 µM), p38 MAPK (p38) inhibitor SB202190 (20 µM), PKA inhibitor H89 (10 µM), PDE inhibitor IBMX (0.1 mM), PI3K inhibitor LY294002 (20 µM), Src kinase Inhibitor I (20 µM), or hedgehog (Hh) inhibitor Jervine (10 µM). After additional incubation for 24 hours, the mRNA expressions of *Epas1*, *Col10a1*, and *Gapdh* were examined by RT-PCR. **B.** At 36 hours after introduction of *Car9* or control siRNA to primary chondrocytes, cAMP was extracted with 0.1 M HCl and its level was determined using an EIA Kit, then corrected for cellular protein. Data are expressed as the mean ± SD of 6 experiments. **C**. At 36 hours after introduction of *Car9* or control siRNA to primary chondrocytes, PKA activity was determined and corrected for cellular protein. Data are expressed as the mean ± SD of 13 determinations. **D-F**. At 24 hours after introduction of *Car9* or control siRNA, chondrocytes were treated for 24 hours with 0.2 mM Br-cAMP. The expression levels of mRNAs for *Car9* (**D**), *Col10a1* (**E**), and *Epas1* (**F**) were analyzed by real-time RT-PCR. Data are expressed as the mean ± SD of 4 experiments. *P*-values determined by two-tailed Mann-Whitney *U*-test are indicated.

To consider the underlying mechanisms of up-regulated expressions of *Epas1* and *Col10a1* in *Car9*-silenced chondrocytes, the effects of several inhibitors of intracellular signaling systems on the expressions of *Col10a1* and *Epas1* were examined in control siRNA- and *Car9* siRNA-introduced chondrocytes (Figure 8A). SP600125, an inhibitor of JNK, did not affect the mRNA expression of *Col10a1* or *Epas1*. SB202190, an inhibitor of p38 MAP kinase (p38 MAPK), augmented the expression of *Epas1* in both *Car9* siRNA- and control siRNA-introduced cells, while its effect on the expression of *Col10a1* was small. H89, an inhibitor of protein kinase A (PKA), up-regulated the expression of *Col10a1* mRNA in both *Car9* siRNA- and control siRNA-introduced cells, while its effect on the expression of *Epas1* mRNA was only marginal. On the other hand, 3-isobutyl-1-methylxanthine (IBMX), an inhibitor of phosphodiesterase (PDE), suppressed augmentation of *Col10a1* expression by *Car9* siRNA. LY294002, a phosphatidylinositol 3-kinase (PI3K) inhibitor, increased the basal expression level of *Epas1* in control siRNA-introduced cells. Also, a Src Kinase Inhibitor I [4-(4’-Phenoxyanilino)-6,7-dimethoxyquinazoline] suppressed the expression of *Col10a1* and *Epas1* in *Car9* siRNA-introduced cells. Jervine, a hedgehog signaling pathway inhibitor, did not show a marked effect on the expression of *Col10a1* or *Epas1* in either *Car9* siRNA- or control siRNA-introduced cells. These results imply that various intracellular signaling systems are involved in expression of *Col10a1* and *Epas1* mRNAs in chondrocytes. Specifically, there is a possibility that p38 MAPK and PI3K are involved in the expression of *Epas1*, and that the cAMP-dependent pathways negatively regulate *Col10a1* expression. In addition, it is also indicated that Src-dependent pathways may be involved in the expression of both *Col10a1* and *Epas1*.

While the effects of H89 and IBMX on *Col10a1* expression suggested an involvement of the cAMP-dependent pathways in induction of *Col10a1* in *Car9*-silenced chondrocytes, cAMP levels in chondrocytes introduced with *Car9* siRNA and its control siRNA were determined at 36 hours after introduction of the siRNAs. The amount of cAMP in the *Car9*-silenced chondrocytes was significantly lower than that in control chondrocytes (Figure 8B, α = 0.05). While a significant difference was not observed, there was a lowering trend in the PKA activity in *Car9*-silenced chondrocytes compared with the control siRNA-introduced cells (Fig. 8C). To further investigate the involvement of the cAMP-dependent pathway in induction of *Col10a1* and *Epas1* expressions after *Car9* silencing, we examined the effects of 8-bromoadenosine-3’,5’-cyclic monophosphate sodium salt (Br-cAMP), a membrane-permeable analog of cAMP, on the expressions of *Col10a1*, *Car9*, and *Epas1* in chondrocytes with or without introduction of *Car9* siRNA. Br-cAMP did not affect the expression of *Car9* in the control or *Car9* siRNA-introduced chondrocytes (Figure 8D). On the other hand, Br-cAMP significantly suppressed the increased expression of *Col10a1* mRNA induced by *Car9* silencing (Figure 8E, α = 0.05), while it up-regulated the expression of *Epas1* mRNA in chondrocytes irrespective of *Car9* expression level (Figure 8F, α = 0.05). These results suggest that the cAMP/PKA pathway is one of the possible mechanisms involved in regulation of *Col10a1* expression by CA IX in chondrocytes.

## Discussion

In the present study, we found that CA IX was distributed among proliferating chondrocytes in mouse epiphyseal cartilage, while its expression was down-regulated in hypertrophic chondrocytes. In addition, introduction of *Car9* siRNA up-regulated the expression of mRNA for *Col10a1* in mouse primary chondrocytes *in vitro*. Taken together, it is reasonable to conclude that CA IX suppresses the expression of *Col10a1*, a typical gene expressed in hypertrophic chondrocytes, both in resting and proliferating chondrocytes. Since the forced expression of CA IX had no effect on proliferation of chondrocytes and expression of *Col10a1* in chondrocytes, there is a possibility that the amount of endogenous CA IX in chondrocytes is sufficient for supporting their proliferation and suppression of *Col10a1* expression. In addition to the change in *Col10a1* expression, lowered expression of *Acan* mRNA (Figure 3G) and reduced Alcian blue binding (Figure 3H) by introduction of *Car9* siRNA, indicating a possibility that CA IX plays a role in regulation of the expression of not only *Col10a1* but also *Acan* gene.

Resting and proliferating chondrocytes in growth plates express HIF-1α [13], whereas hypertrophic chondrocytes express HIF-2α [17]. Replacement of HIF-1α with HIF-2α in growth plates is important for chondrocyte differentiation and skeletal development. HIF-1α binds to the *Sox9* promoter to activate its expression [12]. Since *Sox9* is a master gene of chondrocyte differentiation [18], HIF-1α is required for chondrogenic differentiation of mesenchymal stem cells and maintenance of chondrocyte phenotypes [12], [19]. On the other hand, it is also known that SOX-9 suppresses hypertrophic differentiation of chondrocytes [20], [21], while HIF-2α reportedly plays important roles in hypertrophic differentiation of chondrocytes and endochondral ossification [14], [17]. Transition from HIF-1α to HIF-2α in growth plates has been explained by the differential stability of these HIF proteins. That is, HIF-1α is regarded to be more vulnerable to oxygen than HIF-2α, which causes preferential distribution of the latter in hypertrophic regions which are less hypoxic as compared to resting and proliferating regions of growth plates [10], [22]. Since *Car9* is known to be transactivated by HIF-1α but not HIF-2α [7], [8], it was not unexpected that the expressions of *Car9* mRNA and CA IX protein were observed in proliferating chondrocytes and then down-regulated in hypertrophic chondrocytes (Figures 1 and 2).

HIF-2α is regarded as a regulator of the expression of marker genes of hypertrophic chondrocytes, including *Col10a1*, *Vegfa*, and *Mmp13* [14]. In this study, the expressions of *Col10a1* and *Epas1* were up-regulated by *Car9* siRNA (Figures 3F and 4E), whereas those of *Vegfa* and *Mmp13* were not affected (Figure 3D). In addition, *Epas1* siRNA did not suppress *Car9* siRNA-induced increment in *Col10a1* expression (Figure 7C). These results indicate that *Car9* siRNA induced *Col10a1* transcription through a mechanism independent from the increased expression of *Epas1* mRNA. Lowered expression of HIF-2α protein was confirmed in the chondrocytes introduced with *Epas1* siRNA and those with both *Car9* and *Epas1* siRNAs (Figure 7D), which indicates that the increased expression of *Col10a1* mRNA in *Epas1*-silenced chondrocytes (Figure 7C) was induced by some mechanisms other than HIF-2α. Further studies are required to clarify the mechanisms of *Col10a1* induction in *Epas1*-silenced chondrocytes. Treatment with *Car9* siRNA alone also lowered the expression level of HIF-2α (Figure 7D), even though *Car9* silencing increased the expression level of *Epas1* mRNA (Figure 7B). While there is a possibility that CA IX facilitates translation of the *Epas1* gene or suppresses degradation of HIF-2α protein through an unknown mechanism, further studies are also required to explain the discrepancy between the increased *Epas1* mRNA level and decreased HIF-2α protein level in *Car9*-silenced chondrocytes. At least it can be mentioned that HIF-2α does not play an important role in the induced expression of *Col10a1* mRNA in the *Car9*-silenced chondrocytes.

It has been reported that CA II, one of the isozymes of CA IX, plays a critical role in ameloblast differentiation via intracellular pH-dependent regulation of JNK activity [15]. However, *Car9* siRNA did not induce a significant change in intracellular or extracellular pH in primary chondrocytes in the present experimental settings (Figures 4G and H, α = 0.05). In addition, SP600125, a JNK inhibitor, did not affect the mRNA expression of *Col10a1* (Figure 8A), indicating that CA IX modulates *Col10a1* expression by a mechanism different from that shown in ameloblasts [15].

Among the inhibitors tested in this study, IBMX, an inhibitor of PDE, suppressed the increment in *Col10a1* expression induced by *Car9* siRNA (Figure 8A), suggesting a possible involvement of cyclic nucleotides such as cAMP and/or cGMP in that suppressed expression. Several reports have indicated the role of cGMP-dependent kinase II in promotion of hypertrophic differentiation of chondrocytes [23]–[25]. Therefore, it seems unlikely that cGMP participates in suppression of increased expression of *Col10a1* in *Car9*-silenced chondrocytes by IBMX. Meanwhile it is also known that PKA phosphorylates Runx2 to facilitate its ubiquitination and degradation [26], while PKA was found to phosphorylate SOX9 and enhance its transcriptional activity [27], [28]. RUNX2 is one of the key transcription factors to promote hypertrophic differentiation and SOX9 is known to inhibit hypertrophic differentiation [29]. In the present study, inhibition of PKA augmented the expression of *Col10a1* mRNA, not only in *Car9*-silenced chondrocytes but also in control chondrocytes. Therefore, it is possible that PKA inhibits transcription of the *Col10a1* gene. In addition, our finding of a decrease in cellular level of cAMP in *Car9* siRNA-introduced chondrocytes indicated that CA IX is involved in maintenance of cAMP level in chondrocytes (Figure 8B). Furthermore, Br-cAMP suppressed the augmented expression of *Col10a1* mRNA in chondrocytes harboring *Car9* siRNA (Figure 8E), which reinforces the idea that down-regulation of cAMP-dependent pathways plays a role in induction of *Col10a1* expression in *Car9*-silenced chondrocytes. On the contrary, Br-cAMP enhanced *Epas1* expression in both control and *Car9*-silenced chondrocytes (Figure 8F), indicating that CA IX suppresses *Epas1* expression via a mechanism other than cAMP-dependent pathways.

Several reports have noted biological functions of CA IX aside from its enzymatic activity. For example, it was reported that the proteoglycan-related sequence within the CA IX molecule mediates cell adhesion [30], [31] and that CA IX forms a complex with β-catenin [31]. It is known that the Wnt/β-catenin cascade plays a crucial role in chondrocyte differentiation [25], [32]. In addition, CA IX reportedly functions as a molecular chaperone [33]. Thus, it is suggested that CA IX suppresses the expression of *Col10a1* at least in part via maintenance of the cAMP-dependent pathway in a manner independent of its enzymatic activity resides in the extracellular domain. Further detailed studies are required to clarify how CA IX regulates intracellular cAMP level.

Although more detailed studies on translation, post-translational modifications including phosphorylation, degradation, and molecular interaction of signaling molecules are needed to confirm that CAIX regulates expression of *Col10a1* expression, our present experiments using inhibitors of signaling molecules hint that several intracellular signaling pathways other than cAMP-dependent ones possibly play a part in the expressions of *Col10a1* and *Epas1* mRNAs in chondrocytes. For instance, treatment with a Src kinase inhibitor (Src Kinase Inhibitor I) lowered the expression of *Col10a1* as well as that of *Epas1* in *Car9*-silenced chondrocytes (Figure 8A). It was shown that treatment with PP2, another Src kinase inhibitor, increased the expression of both early and late differentiation markers including *Col10a1* in a chondrogenic cell line ATDC5 [34]. Since it is reported that CA IX interacts with β-catenin [31], one of the substrates of Src kinase [35], there may be an interaction between CA IX and Src in regard to β-catenin. As for *Epas1*, it was reported that Src kinases mediate the hypoxia-induced expression of *EPAS1* mRNA in human lung adenocarcinoma cells [36]. On the other hand, inhibition of p38 MAPK and PI3K increased the expression of *Epas1* in the control chondrocytes without *Car9* silencing in our experiments (Figure 8A). Although the requirement of MEK/ERK pathways for *EPAS1* expression under hypoxia has been reported [37], to the best of our knowledge, the role of p38 MAPK or PI3K in regulation of *Epas1* expression have not been studied.

**Figure 9:**
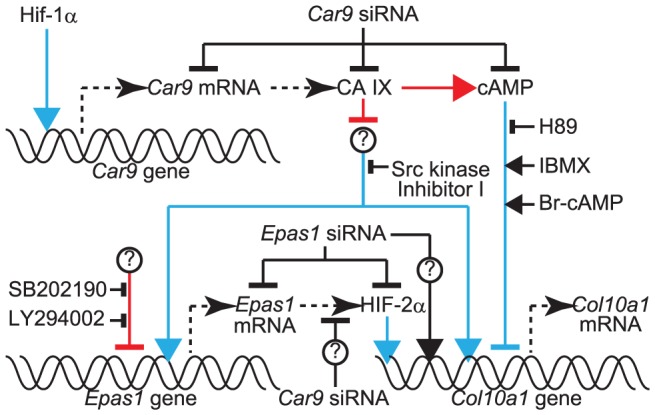
Schematic representation of possible roles of CA IX in regulation of *Col10a1* expression in chondrocytes. Blue and red lines represent pathways already known and those indicated in this study, respectively. Arrows with equilateral-triangular heads and T-shaped bars show facilitation and suppression of the pathways, respectively. HIF-1α induces the expression of *Car9* mRNA and hence that of CA IX protein [8]. It is known that cAMP-dependent pathways inhibit hypertrophic differentiation of chondrocytes including the expression of *Col10a1* [26]-[29]. On the other hand, it is reported that HIF-2α, encoded by *Epas1* gene, transactivates the *Col10a1* gene [14]. We propose that CA IX suppresses the expression of *Col10a1* mRNA partly via a cAMP-dependent manner based on the following observations. *Col10a1* expression was induced by introduction of *Car9* siRNA (Figure 3G). The cAMP level was lowered by *Car9* siRNA (Figure 8B). Inhibition of PKA by H89 augmented the expression of *Col10a1* induced by *Car9* silencing (Figure 8A). In addition, inhibition of PDE by IBMX and activation of PKA by Br-cAMP suppressed the expression of *Col10a1* expression induced by *Car9* silencing (Figures 8A and 8D). While *Car9* siRNA also enhanced the expression of *Epas1* mRNA (Figure 4E), HIF-2α does not mediate the *Col10a1* induction by *Car9* silencing (Figure 7C). It is partly because *Car9* siRNA lowers HIF-2α protein via an unknown mechanism (Figure 7D). *Epas1* siRNA rather enhanced the expression of *Col10a1* mRNA by an unknown mechanism (Figure 7C). It is reported that Src kinase mediates the expression of *Epas1* and *Col10a1* [34], [36]. In this study, Src kinase inhibitor I lowered the induction of *Epas1* and *Col10a1* expressions after *Car9* silencing (Figure 8A), which may indicate a possibility that CA IX suppresses the Src kinase-mediated pathways. Augmentation of *Epas1* expression in the presence of the inhibitors of p38 MAPK (SB202190) and PI3K (LY294002) suggests a possible inhibition of *Epas1* expression by these kinases (Figure 8A).

From the results obtained in this study, we propose a novel role for CA IX in cartilage development that is down-regulating *Col10a1* mRNA expression in stationary and proliferating chondrocytes (Figure 9). Hence, down-regulation of CA IX in hypertrophic regions of growth plates triggers *Col10a1* expression. On the other hand, the forced expression of CA IX had no effect on the expression of *Col10a1* in chondrocytes, suggesting a possibility that the amount of endogenous CA IX in chondrocytes is sufficient for suppression of *Col10a1* expression.

## Materials and Methods

### Ethics Statement

All animal experiments were approved by the Ethical Board for Animal Experiments of Showa University (Approval No. 11027), Tokyo, Japan.

### Mice

We used ddY mice provided by Japan SLC Inc. (Hamamatsu, Japan). They were housed in a specific pathogen-free environment.

### Preparation of Frozen Fixed Samples

Lower limbs were resected from 1-day-old postnatal ddY mice, embedded in OCT compound (Sakura Finetechnical Co. Ltd., Tokyo, Japan), and immediately snap frozen in isopentane cooled in liquid nitrogen. The specimens were then made into frozen blocks and stored at -80°C. Frozen samples prepared for laser microdissection were sliced using a cryomicrotome (MICROM International GmbH, Walldorf, Germany) at a thickness of 7 µm, then each tissue section was affixed to a slide to which an original thin film (provided by Meiwa Shoji Co., Ltd., Tokyo, Japan) had been attached using silicone adhesive (GE Toshiba Silicone, Tokyo, Japan). Frozen tissues for immunohistochemistry were sliced at a thickness of 4 µm. Sliced samples were stored at -40°C until use.

### Laser Microdissection and cDNA Preparation from RNA Isolated from Micro-dissected Samples

The sliced samples were air-dried at room temperature for 2 to 3 minutes and fixed in 100% methanol for 3 minutes. The sections were then washed with 50% ethanol, stained with LCM Staining Kit (Ambion, Carlsbad, CA, USA), and washed with 100% ethanol, then air-dried, after which they were subjected to microdissection using a PALM Micro Beam (P.A.L.M. Microlaser Technologies, Bernried, Germany) with a 337-nm nitrogen laser. The targets were regions that mainly consisted of round proliferating chondrocytes, columnar proliferating chondrocytes with pre-hypertrophic chondrocytes, and hypertrophic chondrocytes (Figure 2A). Each region was collected from 4 serial sections. There are estimated to be approximately 500–1000 microdissected cells per region. Total RNA was extracted from the laser micro-dissected tissues using an RNeasy Micro Kit (Qiagen, Valencia, CA, USA). At the final step of extraction, RNA was eluted in 17 µl of DNase/RNase-free water (Invitrogen Co., Carlsbad, CA, USA). RT reaction was performed using High Capacity RNA-to cDNA MasterMix (Applied Biosystems, Carlsbad, CA, USA) with 16 µl of eluted RNA solution.

### Immunohistochemistry of Type II Collagen

Frozen sections at 4 µm thick were air-dried for 10 minutes, fixed by incubation for 5 minutes in ice-cold acetone, and washed 3 times in phosphate-buffered saline (PBS). Endogenous peroxidase activity was blocked by treatment for 30 minutes in methanol containing 1% hydrogen peroxide. The samples were then treated for 15 minutes with Protein Block Serum-Free Ready-to-Use (DAKO, Tokyo, Japan) before incubation for 1 hour at 37°C with anti-chicken type II collagen monoclonal IgG (10R-C135A, Fitzgerald Industries International, Acton, MA, USA) at a dilution ratio of 1:100. After washing 3 times with PBS, the primary antibody bound to the samples was visualized by treatment for 30 minutes at room temperature with DAKO EnVision^+TM^ System-HRP Labeled Polymer Anti-Mouse (K4000, DAKO) and subsequent incubation with a DAB substrate kit (K3468, DAKO). After washing with PBS, the slides were observed under a light microscope.

### Immunohistochemistry of Type X Collagen

Frozen sections at 4 µm thick were air-dried for 10 minutes, fixed by incubation for 5 minutes in ice-cold ethanol, and washed 3 times in PBS. Endogenous peroxidase activity was blocked by treatment for 10 minutes in methanol containing 3% hydrogen peroxide. The samples were treated for 10 minutes at 43°C with 0.5% bovine serum albumin in PBS, washed 3 times with PBS, and incubated for 30 minutes at room temperature in PBS containing 2 mg/ml hyaluronidase from bovine testes (Sigma-Aldrich). After stopping the hyaluronidase activity with fetal bovine serum (FBS) (Invitrogen), the samples were incubated for 45 minutes at 37°C with anti-rat type X collagen rabbit polyclonal antibody (No. 234196, Calbiochem, Darmstadt, Germany) at a dilution ratio of 1:50 and washed 3 times with PBS. The primary antibody was visualized following treatment for 30 minutes at room temperature with DAKO EnVision^+TM^ System-HRP Labeled Polymer Anti-Rabbit (K4003, DAKO) and subsequent incubation with a DAB substrate kit (DAKO). After washing with PBS, the slides were observed under a light microscope.

### Immunohistochemistry of CA IX

Frozen sections at 4 µm thick were air-dried for 10 minutes, fixed by incubation for 10 minutes in 4% paraformaldehyde, pH 7.4, and washed 3 times in PBS. After blocking the endogenous peroxidase activity by 3% hydrogen peroxide, the samples were treated with Protein Block Serum-Free Ready-to Use (DAKO). The samples were then incubated for 1 hour at room temperature with anti-mouse CA IX antibody (M-100 anti-mouse CA IX rabbit polyclonal antibody, sc-25600, Santa Cruz Biotechnology, Inc., Santa Cruz, CA, USA). After washing with PBS, the primary antibody was visualized following treatment for 30 minutes at room temperature with DAKO EnVision^+TM^ System-HRP Labeled Polymer Anti-Rabbit (DAKO) and subsequent incubation with a DAB substrate kit (DAKO). After washing with PBS, the slides were observed under a light microscope.

### Cell Culture

Primary chondrocytes were isolated from costal cartilages of 1-day postnatal ddY mice (Japan SLC). Briefly, rib cages were excised from the mice and digested to remove soft tissues for 1 hour at 37°C with 1 mg/ml collagenase A (Roche Diagnostics GmbH, Mannheim, Germany) in a mixture of Dulbecco’s modified Eagle’s medium and Ham F12 medium (DMEM/F12), containing antibiotics and 2.5% FBS. Cartilage specimens were washed with PBS and further digested for 15 hours at 37°C in the same medium containing 1 mg/ml collagenase A. The resultant cell suspension was filtered through a 70-µm cell strainer to remove debris. Primary chondrocytes were washed by centrifugation and used in the experiments without passage. Experimental conditions for introduction of the siRNAs and the plasmids into and cultivation of chondrocytes were described below. For cell proliferation assay, chondrocytes were plated in 96-well plates at a density of 1×10^4^ cell/well. Cell proliferation was assessed spectrophotometrically using CellTiter 96® Aqueous One Solution (Promega, Madison, WI, USA), according to the manufacturer’s instructions. In some experiments, isolated chondrocytes were cultured under a hypoxic condition (5% CO_2_ and 95% N_2_) using an INVIVO2 hypoxia workstation (Ruskinn Technology Ltd., Bridgend, UK).

### Introduction of siRNAs for *Car9* and *Epas1* into Chondrocytes

Stealth^TM^ siRNAs for mouse *Car9* and *Epas1* and their control non-silencing siRNA were purchased from Invitrogen. The sense and antisense sequences of Stealth^TM^ siRNAs for *Car9* and *Epas1*, designed using BLOCK-iT^TM^ RNAi Designer (Invitrogen), are shown in Table S1. Each siRNA (30 pmol) was introduced into 40-50% confluent chondrocytes cultured in antibiotics-free DMEM/F12 containing 2.5% FBS in 6-well plates using Lipofectamine RNAiMAX (Invitrogen) by reverse transfection under 5% CO_2_–95% air (a normoxic condition) or 5% CO_2_–95% N_2_ (a hypoxic condition). After 24 hours of incubation, the culture medium was changed to fresh DMEM/F12 + 2.5% FBS containing antibiotics. In some experiments, *Epas1* siRNA (30 pmol) and *Car9* siRNA (30 pmol) were simultaneously introduced into chondrocytes in the same manner as described above. Expression of *Car9* and *Epas1* mRNAs was evaluated by RT-PCR and real-time RT-PCR, as described below.

### Immunological evaluation of CA IX knockdown

CA IX protein level in *Car9*-silenced cells was visualized by immunocytochemistry. Briefly, primary chondrocytes introduced with *Car9* siRNA or its control siRNA were fixed by treatment with 4% paraformaldehyde (pH 7.4) for 10 minutes at 4°C, then incubated for 1 hour with a 4% solution of Block Ace^TM^ (Dainippon Pharma, Osaka, Japan). Next, the cells were incubated overnight at 4°C with 0.5 µg/ml M-100 anti-mouse CA IX rabbit polyclonal antibody (Santa Cruz) in 0.4% Block Ace solution. The cell-bound primary antibody was visualized under a fluorescence microscope after incubation with Alexa Fluor® 488 donkey anti-rabbit IgG (Invitrogen).

### Forced Expression of CA IX in Chondrocytes

An expression plasmid for CA IX was prepared by insertion of a RIKEN Mouse FANTOM® clone of *Car9* cDNA (clone ID 9130714F03, GenBank accession AK136579.1) purchased from DNAFORM (Yokohama, Japan) into pcDNA^TM^6/His C (Invitrogen). The CA IX expression plasmid and its control plasmid (pcDNA^TM^6/His C) (4 µg) were transfected into 40–50% confluent chondrocytes cultured in 6-well plates using Lipofectamine 2000 (Invitrogen). Forced expression of CA IX protein was confirmed by Western blot analysis.

### Western Blot Analysis

Cells were lysed in 10 mM Tris-HCl (pH 7.8) containing 1% NP-40, 0.15 M NaCl, and a protease inhibitor cocktail (Roche Diagnostics, Manheim, Germany). The cell lysates (5 µg protein) were subjected to SDS-PAGE (10% polyacrylamide gel) under a reducing condition. Following electrophoresis, proteins were transferred onto polyvinylidene difluoride membranes. The membranes were incubated overnight at 4°C with M-100 anti-mouse CA IX rabbit polyclonal antibody (Santa Cruz Biotechnology) (dilution ratio, 1:1,000) or anti-HIF-2α rabbit polyclonal antibody (Cat. No. NB100–122, Novus Biologicals, Littleton, CO, USA) (dilution ratio, 1:1,000), and then with anti-rabbit IgG linked with horseradish peroxidase (GE Healthcare, Waukesha, WI) (dilution ratio, 1:10,000). Immunoreactive bands were visualized using an ECL plus system (GE Healthcare).

### Treatment of Chondrocytes with Chemicals

Br-cAMP (Cat. No. B5386), a membrane-permeable derivative of cAMP, 4-(2-aminoethyl)benzenesulfonamide (Cat. No. 275247), an inhibitor of extracellular CAs, IBMX (Cat. No. I5879), a PDE inhibitor, SP600125 (Cat. No. S5567), a JNK inhibitor, and LY294002 (Cat. No. L9908), a PI3K inhibitor, were purchased from Sigma-Aldrich. Src Kinase Inhibitor I (Cat. No. 567805), SB202190 (Cat. No. 559388), a p38MAPK inhibitor, H89 (Cat. No. 371963), a PKA inhibitor, and Jervine (Cat. No. 420210), a hedgehog signaling pathway inhibitor, were obtained from Calbiochem (La Jolla, CA, USA). These inhibitors were added to cultures at 24 hours after introduction of the siRNAs and the cells were cultured for an additional 24 hours in their presence. The concentrations of the reagents in culture medium were as follows: Br-cAMP, 0.2 mM; 4-(2-aminoethyl)benzenesulfonamide, 50 µM; IBMX, 0.1 mM; SP600125, 30 µM; LY294002, 20 µMl; Src kinase Inhibitor I, 20 µM; SB202190, 20 µM; H89, 10 µM; and Jervine, 10 µM.

### Determination of cAMP Content in Chondrocytes

cAMP was extracted from the cultured chondrocytes with 0.1 M HCl at 36 hours after introduction of *Car9* and control siRNAs. cAMP content was quantified using a Cyclic AMP EIA Kit (Cayman Chemical Co., Ann Arbor, MI, USA), according to the manufacturer’s instructions. The cAMP content was corrected for cellular protein determined using a BCA^TM^ Protein Assay Kit (Thermo Scientific, Rockford, IL, USA).

### Measurement of PKA Activity in Chondrocytes

PKA activity in the lysates of chondrocytes was determined using a PKA Kinase Activity assay kit (Enzo Life Sciences, Farmingdale, NY, USA) according to the manufacturer’s instruction at 36 hours after introduction of *Car9* siRNA or control siRNA. The active PKA content was corrected for cellular protein.

### RNA Extraction from Cells and RT Reaction

Total RNA was extracted from cells using TRIzol® reagent (Life Technologies, Carlsbad, CA) according to the manufacturer’s instructions. Quality of RNA was checked by agarose-gel electrophoresis. Concentration and quality of RNA were analyzed using NanoDrop® Spectrophotometer ND-1000 (Thermo Fisher Scientific Inc., Waltham, MA, USA). Fresh RNA samples having the ratio of absorptions at 260 nm vs. 280 nm (260/280) higher than 1.8 were applied to RT reactions. cDNA was synthesized in a 10 µl reaction mixture containing 2 µg of total RNA using Superscript® III reverse transcriptase (Invitrogen) and a random hexamer (Invitrogen) following the manufacturer’s instructions. Since amplification was not detected in the PCR reactions using RT reaction products obtained in the absence of reverse transcriptase, we did not treat the RNA samples with DNase.

### PCR Analysis

cDNA samples obtained as described above were diluted to 1:5 with DNase/RNase-free water (Invitrogen).Each diluted cDNA sample (2 µl) was added to a total of 20 µl reaction mixture containing GoTaq® Green Master Mix (Promega) and appropriate primers. The PCR primers, synthesized and supplied by Invitrogen, were designed using Primer3 software (http://primer3.sourceforge.net/), based on the reported sequences of their mRNAs. The sequences of the primers, amplification sites, and accession numbers of *Car9*, *Col2a1*, *Col10a1*, *Acan*, *Mmp13*, *Vegfa*, *Hif1a*, *Epas1*, *Hif3a*, and glyceraldehyde-3-phosphate dehydrogenase (*Gapdh*) are shown in Table S2. The PCR products were separated on 1.0% agarose gels and stained with ethidium bromide.

### Quantitative Real Time PCR Analysis

Quantitative real time PCR was performed and reported according to the MIQE guidelines [39]. cDNA samples were diluted to 1:10 with DNase/RNase-free water. Quantitative real time PCR analyses were performed using TaqMan® Gene Fast Universal PCR Master Mix (Applied Biosystems) with a StepOne® Real-Time PCR System (Applied Biosystems). The program used was StepOne® Software (ver. 2.0, Applied Biosystems). The total reaction volume was 10 µl including 2 µl of the diluted cDNA. The thermo-cycling parameters employed were as follows: holding for 20 seconds at 95°C, followed by 40 cycles of a denaturation at 95°C for 1 second and annealing and elongation at 60°C for 20 seconds. The probe and primer pairs for *Car9*, *Col2a1*, *Acan*, *Col10a1*, *Sox5*, *Sox6*, *Sox9*, *Epas1*, and *Gapdh* were supplied by Applied Biosystems. Their assay numbers were listed in Table S3. All real time PCR runs were accompanied by a dilution series to calibrate PCRs for empirical efficiency. The expression level of each gene was normalized against that of *Gapdh* and expressed as the relative value for each experiment. *Gapdh* is one of the widely-used reference genes. In this study, C_t_ values of *Gapdh* were stable based on the amounts of mRNA among different total RNA samples extracted from chondrocytes introduced with *Car9* siRNA and its control siRNA. Additional information about real time PCR performed in this study is summarized in Table S4.

### Measurements of Intracellular and Extracellular pH

The pH value of the culture medium samples was determined as extracellular pH using a pH electrode (Horiba Ltd., Kyoto, Japan). Intracellular pH was determined as described previously [38]. Briefly, primary chondrocytes were detached by collagenase digestion, washed with PBS, resuspended in HEPES-buffered saline containing 5 mM glucose (HBSG) (pH 7.4), and treated for 30 minutes with 10 µM BCECF-AM (Dojindo Laboratories, Kumamoto, Japan). After washing with HBSG, cells were resuspended in HBSG and the fluorescence intensity of BCECF in the cells was measured using a Hitachi F-4000 fluorescence spectrophotometer (Hitachi Ltd., Tokyo, Japan). The cells were alternately excited at 455 or 505 nm with a 150 W xenon lamp and fluorescence emission was monitored at 530 nm at intervals of 5 seconds. The 505/455 excitation ratio corresponded to a specific intracellular pH. Calibration of pH was performed using cells permeabilized in buffers containing 10 µM nigericin (N7143, Sigma-Aldrich), 130 mM KCl, 10 mM NaCl, 1 mM MgSO_4_, and 10 mM MOPS, with various pH values.

### Statistical Analysis

Online software programs were used for statistical analyses [40], [41]. Nonparametric Mann Whitney *U*-test was used for comparisons between the results from 2 groups. Steel-Dwass test, a non-parametric post-hoc test, was used for all the pairwise comparisons of the results from 3 or more groups, The *P*-values were shown in the figures. The values used for drawing figures and statistical analyses employed in this study are listed in Tables S5 and S6, respectively.

## Supporting Information

Table S1 **Sense and antisense sequences of Stealth^TM^ siRNAs for** ***Car9*** **and** ***Epas1*** **.**
(DOC)

Table S2 **Primers for RT-PCR.**
(DOC)

Table S3 **TaqMan® probes used in this study.** See details at the Web site of TaqMan® Assays (http://www.invitrogen.com/site/us/en/home/Products-and-Services/Applications/PCR/real-time-pcr/real-time-pcr-assays/taqman-gene-expression.html?s_kwcid=TC|13009|taqman%20probe||S|b|11989269653).
(DOC)

Table S4 **Real time PCR quality control.**
(DOC)

Table S5 **List of the data analyzed statistically in this study.**
(DOC)

Table S6 **Statistical analysis performed for drawing the Figures.**
(DOC)
