# Supplementary material for: Downregulation of Carbonic Anhydrase IX Promotes Col10a1 Expression in Chondrocytes
Source: PLoS One. 2013 Feb 18;8(2):e56984. doi: 10.1371/journal.pone.0056984 (PMC3575511; doi:10.1371/journal.pone.0056984)
Supplement: Table S1 — Sense and antisense sequences of StealthTM siRNAs for Car9 and Epas1 . (DOC) [file pone.0056984.s001.doc]

**Table S1. Sense and antisense sequences of Stealth^TM^ siRNAs for *Car9* and *Epas1*.**

| Gene |  | Sequence | Accession |
| --- | --- | --- | --- |
| *Car9* | Sense | 5’-CAG UAC UGC UUU CUC CGA ACU UCA U-3’ | AJ245857 |
|  | Antisense | 5’-AUG AAG UUC GGA GAA AGC AGU ACU G-3’ |  |
| *Epas1* | Sense | 5’-CCA UCA GCU UCC UUC GGA CAC AUA A-3’ | NM_010137 |
|  | Antisense | 5’-UUA UGU GUC CGA AGG AAG CUG AUG G-3’ |  |
