# Supplementary material for: Downregulation of Carbonic Anhydrase IX Promotes Col10a1 Expression in Chondrocytes
Source: PLoS One. 2013 Feb 18;8(2):e56984. doi: 10.1371/journal.pone.0056984 (PMC3575511; doi:10.1371/journal.pone.0056984)
Supplement: Table S2 — Primers for RT-PCR. (DOC) [file pone.0056984.s002.doc]

**Table S2. Primers for RT-PCR.**

| Gene | Primer | | Accession | Position | Amplicon size (bp) |
| --- | --- | --- | --- | --- | --- |
| *Car9* | Sense | 5’-TTC AGT CCC CGG TAG ACA TC-3’ | AJ245857 | 448-864 | 417 |
|  | Antisense | 5’-TTT CTT CCA AAT GGG ACA GC-3’ |  |  |  |
| *Col2a1* | Sense | 5’-GCC AAG ACC TGA AAC TCT GC-3’ | NM_001113515 | 3875-4368 | 494 |
|  | Antisense | 5’-CTT GCC CCA CTT ACC AGT GT-3’ |  |  |  |
| *Col10a1* | Sense | 5’-CCA CCT GGG TTA GAT GGA AAA-3’ | NM_009925 | 1219-1888 | 670 |
|  | Antisense | 5’-AAT CTC ATC AAA TGG GAT GGG-3’ |  |  |  |
| *Acan* | Sense | 5’-CAG GTT TCC CCA CTG TGT CT-3’ | NM_007424 | 4833-5319 | 487 |
|  | Antisense | 5’-ACT CCA GAC CCT GGG AAG TT-3’ |  |  |  |
| *Mmp13* | Sense | 5’-AGT TGA CAG GCT CCG AGA AA-3’ | BC125320 | 236-748 | 513 |
|  | Antisense | 5’-TCC TTG GAG TGA TCC AGA CC-3’ |  |  |  |
| *Vegfa* | Sense | 5’-GCC CTG AGT CAA GAG GAC AG-3’ | BC061468 | 1606-2037 | 432 |
|  | Antisense | 5’-GGA AGG GAA GAT GAG GAA GG-3’ |  |  |  |
| *Hif1a* | Sense | 5’-TGC TCA TCA GTT GCC ACT TC-3’ | AF003695 | 377-827 | 451 |
|  | Antisense | 5’-CTT CCA CGT TGC TGA CTT GA-3’ |  |  |  |
| *Epas1* | Sense | 5’-CCA GCA CTG CTT CAG TAC CA-3’ | NM_010137 | 2096-2596 | 501 |
|  | Antisense | 5’-GTG TCT TGG AAG GCT TGC TC-3’ |  |  |  |
| *Hif3a* | Sense | 5’-TAC ACG GAG ACC CCA AAG TC-3’ | AF060194 | 1379-1842 | 464 |
|  | Antisense | 5’-GCT TGG GAG GCT TAA TTT CC-3’ |  |  |  |
| *Gapdh* | Sense | 5’-ACC ACA GTC CAT GCC ATG AC-3’ | NM_008084 | 570-1021 | 452 |
|  | Antisense | 5’-TCC ACC ACC CTG TTG CTG TA-3’ |  |  |  |
