# Supplementary material for: Downregulation of Carbonic Anhydrase IX Promotes Col10a1 Expression in Chondrocytes
Source: PLoS One. 2013 Feb 18;8(2):e56984. doi: 10.1371/journal.pone.0056984 (PMC3575511; doi:10.1371/journal.pone.0056984)
Supplement: Table S3 — TaqMan® probes used in this study. See details at the Web site of TaqMan® Assays (http://www.invitrogen.com/site/us/en/home/Products-and-Services/Applications/PCR/real-time-pcr/real-time-pcr-assays/taqman-gene-expression.html?s_kwcid=TC|13009|taqman%20probe||S|b|11989269653). (DOC) [file pone.0056984.s003.doc]

**Table S3. TaqMan**® **probes used in this study.**

See details at the Web site of TaqMan® Assays (http://www.invitrogen.com/site/us/en/home/Products-and-Services/Applications/PCR/real-time-pcr/real-time-pcr-assays/taqman-gene-expression.html?s_kwcid=TC|13009|taqman%20probe||S|b|11989269653).

| Gene | Assay ID | Exon boundary | Probe spans exon | Location | Amplicon size | Reference Sequence |
| --- | --- | --- | --- | --- | --- | --- |
| *Car9* | Mm00519870_m1 | 5 and 6 | Yes | 808 | 70 | NM_139305.2 |
| *Col2a1* | Mm01309562_g1 | 52 and 53 | Yes | 4345 | 71 | NM_001113515.2 |
| *Acan* | Mm00545807_m1 | 15 and 16 | Yes | 6275 | 78 | NM_007424.2 |
| *Col10a1* | Mm00487041_m1 | 2 and 3 | Yes | 223 | 77 | NM_009925.4 |
| *Sox5* | Mm00488381_m1 | 8 and 9 | Yes | 1209 | 60 | NM_011444.2 |
| *Sox6* | Mm00488393_m1 | 8 and 9 | Yes | 1041 | 94 | NM_001025559.2 |
| *Sox9* | Mm03003574_s1 | 1 | No | 592 | 96 | NM_011448.4 |
| *Epas1* | Mm01236112_m1 | 6 and 7 | Yes | 1192 | 63 | NM_010137.3 |
| *Gapdh* | Mm03302249_g1 | 1 | No | 1000 | 70 | NM_008084.2 |
