# Supplementary material for: Downregulation of Carbonic Anhydrase IX Promotes Col10a1 Expression in Chondrocytes
Source: PLoS One. 2013 Feb 18;8(2):e56984. doi: 10.1371/journal.pone.0056984 (PMC3575511; doi:10.1371/journal.pone.0056984)
Supplement: Table S4 — Real time PCR quality control. (DOC) [file pone.0056984.s004.doc]

**Table S4. Real time PCR quality control.**

| Gene | PCR efficiency | Linear dynamic range | r^2^ | Slope | Y intersept | C_q_ at LOD |
| --- | --- | --- | --- | --- | --- | --- |
| *Car9* | 94.931% | 27.34-36.84 | 0.995 | -3.450 | 27.509 | 22.56 |
| *Col2a1* | 98.740% | 19.72-28.30 | 0.994 | -3.245 | 16.524 | 8.35 |
| *Acan* | 103.309% | 16.45-21.44 | 0.999 | -3.352 | 19.683 | 12.26 |
| *Col10a1* | 99.586% | 26.73-34.12 | 0.995 | -3.332 | 26.955 | 22.89 |
| *Sox5* | 110.523% | 27.16-36.21 | 0.998 | -3.093 | 33.402 | 19.60 |
| *Sox6* | 113.572% | 24.52-31.66 | 0.999 | -3.034 | 30.617 | 17.47 |
| *Sox9* | 123.708% | 25.15-33.09 | 0.994 | -2.859 | 31.126 | 19.76 |
| *Epas1* | 95.910% | 29.88-38.36 | 0.999 | -3.424 | 29.879 | 23.32 |
| *Gapdh* | 95.270% | 17.67-25.83 | 1.000 | -3.441 | 17.712 | 13.33 |
