# Supplementary material for: Downregulation of Carbonic Anhydrase IX Promotes Col10a1 Expression in Chondrocytes
Source: PLoS One. 2013 Feb 18;8(2):e56984. doi: 10.1371/journal.pone.0056984 (PMC3575511; doi:10.1371/journal.pone.0056984)
Supplement: Table S5 — List of the data analyzed statistically in this study. (DOC) [file pone.0056984.s005.doc]

**Table S5. List of the data analyzed statistically in this study.**

| Figure | Subject | Sample | Sample size | Mean | SD |
| --- | --- | --- | --- | --- | --- |
| 2B | *Col2a1* mRNA | Site 1 | 6 | 1 | 0.209 |
|  |  | Site 2 | 6 | 0.455 | 0.381 |
|  |  | Site 3 | 6 | 0.132 | 0.0418 |
| 2C | *Col10a1* mRNA | Site 1 | 6 | 1 | 2.168 |
|  |  | Site 2 | 6 | 1.7 | 1.61 |
|  |  | Site 3 | 6 | 21.7 | 6.82 |
| 2D | *Car9* mRNA | Site 1 | 6 | 1 | 0.363 |
|  |  | Site 2 | 6 | 0.213 | 0.258 |
|  |  | Site 3 | 6 | 0.0336 | 0.0449 |
| 3A | *Car9* mRNA | Control | 4 | 1 | 0.09 |
|  |  | *Car9* siRNA | 4 | 0.225 | 0.047 |
| 3B | Cell proliferation | Control Day 0 | 4 | 1.101 | 0.125 |
|  |  | *Car9* siRNA Day 0 | 4 | 0.918 | 0.099 |
|  |  | Control Day 2 | 4 | 1.025 | 0.038 |
|  |  | *Car9* siRNA Day 2 | 4 | 0.872 | 0.059 |
|  |  | Control Day 4 | 4 | 1.522 | 0.0518 |
|  |  | *Car9* siRNA Day 4 | 4 | 1.29 | 0.107 |
|  |  | Control Day 7 | 4 | 1.857 | 0.185 |
|  |  | *Car9* siRNA Day 7 | 4 | 1.991 | 0.089 |
|  |  | Control Day 10 | 4 | 2.021 | 0.084 |
|  |  | *Car9* siRNA Day 10 | 4 | 1.635 | 0.098 |
| 3E | *Col2a1* mRNA | Control | 4 | 1 | 0.235 |
|  |  | *Car9* siRNA | 4 | 0.753 | 0.058 |
| 3F | *Col10a1* mRNA | Control | 4 | 1 | 0.078 |
|  |  | *Car9* siRNA | 4 | 7.98 | 1.39 |
| 3G | *Acan* mRNA | Control | 4 | 1 | 0.08 |
|  |  | *Car9* siRNA | 4 | 0.488 | 0.028 |
| 3H | Alcian blue | Control | 4 | 0.0643 | 0.0035 |
|  |  | *Car9* siRNA | 4 | 0.0453 | 0.0048 |

**Table S5. List of the data analyzed statistically in this study (contimued).**

| Figure | *Subject* | Sample | Sample size | Mean | SD |
| --- | --- | --- | --- | --- | --- |
| 4A | *Sox5* mRNA | Control | 4 | 1 | 0.3 |
|  |  | *Car9* siRNA | 4 | 1.29 | 0.495 |
| 4B | *Sox6* mRNA | Control | 4 | 1 | 0.085 |
|  |  | *Car9* siRNA | 4 | 1.26 | 0.428 |
| 4C | *Sox9* mRNA | Control | 4 | 1 | 0.271 |
|  |  | *Car9* siRNA | 4 | 1.57 | 0.497 |
| 4D | *Epas1* mRNA | Control | 4 | 1 | 0.159 |
|  |  | *Car9* siRNA | 4 | 3.67 | 0.625 |
| 4G | Intracellular pH | Control | 4 | 7.49 | 0.045 |
|  |  | *Car9* siRNA | 4 | 7.5 | 0.088 |
| 4H | Extracellular pH | Control | 4 | 7.3 | 0.033 |
|  |  | *Car9* siRNA | 4 | 7.32 | 0.017 |
| 5A | *Car9* mRNA | Control | 4 | 1 | 0.256 |
|  |  | *Car9* siRNA | 4 | 0.261 | 0.069 |
| 5B | *Col10a1* mRNA | Control | 4 | 1 | 0.124 |
|  |  | *Car9* siRNA | 4 | 2.359 | 0.554 |
| 5C | *Epas1* mRNA | Control | 4 | 1 | 0.224 |
|  |  | *Car9* siRNA | 4 | 2.437 | 0.303 |
| 5D | *Sox5* mRNA | Control | 4 | 1 | 0.05 |
|  |  | *Car9* siRNA | 4 | 1.515 | 0.053 |
| 5E | *Sox6* mRNA | Control | 4 | 1 | 0.029 |
|  |  | *Car9* siRNA | 4 | 1.309 | 0.169 |
| 5F | *Sox9* mRNA | Control | 4 | 1 | 0.237 |
|  |  | *Car9* siRNA | 4 | 1.743 | 0.255 |

**Table S5. List of the data analyzed statistically in this study (contimued).**

| Figure | Subject | Sample | Sample size | Mean | SD |
| --- | --- | --- | --- | --- | --- |

| 6B | Cell proliferation | Control (day 0) | 4 | 0.555 | 0.038 |
| --- | --- | --- | --- | --- | --- |
|  |  | *Car9* OE (day 0) | 4 | 0.58 | 0.108 |
|  |  | Control (day 2) | 4 | 0.722 | 0.094 |
|  |  | *Car9* OE (day 2) | 4 | 0.716 | 0.107 |
|  |  | Control (day 4) | 4 | 1.067 | 0.156 |
|  |  | *Car9* OE (day 4) | 4 | 1.102 | 0.042 |
|  |  | Control (day 7) | 4 | 1.697 | 0.075 |
|  |  | *Car9* OE (day 7) | 4 | 1.589 | 0.066 |
|  |  | Control (day 10) | 4 | 1.638 | 0.061 |
|  |  | *Car9* OE (day 10) | 4 | 1.559 | 0.091 |
| 6C | *Col2a1* mRNA | Control | 4 | 1 | 0.127 |
|  |  | *Car9* OE | 4 | 1.175 | 0.102 |
| 6D | *Acan* mRNA | Control | 4 | 1 | 0.104 |
|  |  | *Car9* OE | 4 | 1.289 | 0.054 |
| 6E | *Col10a1* mRNA | Control | 4 | 1 | 0.367 |
|  |  | *Car9* OE | 4 | 0.989 | 0.443 |
| 6F | *Epas1* mRNA | Control | 4 | 1 | 0.323 |
|  |  | *Car9* OE | 4 | 0.892 | 0.243 |
| 7A | *Car9* mRNA | Control/Control | 5 | 1 | 0.15 |
|  |  | si*Car9*/Control | 5 | 0.262 | 0.03 |
|  |  | Control/si*Epsa1* | 5 | 0.731 | 0.049 |
|  |  | si*Car9*/si*Epas1* | 5 | 0.371 | 0.031 |
| 7B | *Epas1* mRNA | Control/Control | 5 | 1 | 0.19 |
|  |  | si*Car9*/Control | 5 | 1.963 | 0.559 |
|  |  | Control/si*Epsa1* | 5 | 0.595 | 0.111 |
|  |  | si*Car9*/si*Epas1* | 5 | 0.758 | 0.164 |
| 7C | *Col10a1* mRNA | Control/Control | 5 | 1 | 0.582 |
|  |  | si*Car9*/Control | 5 | 4.999 | 0.601 |
|  |  | Control/si*Epsa1* | 5 | 2.924 | 0.305 |
|  |  | si*Car9*/si*Epas1* | 5 | 3.913 | 0.547 |

**Table S5. List of the data analyzed statistically in this study (contimued).**

| 8B | cAMP | Control | 6 | 51.5 | 2.8 |
| --- | --- | --- | --- | --- | --- |
|  |  | *Car9* siRNA | 6 | 39.6 | 8.2 |
| 8C | *Car9* mRNA | Control/Control | 4 | 1 | 0.154 |
|  |  | Control/Br-cAMP | 4 | 0.794 | 0.152 |
|  |  | *Car9* siRNA/Control | 4 | 0.134 | 0.134 |
|  |  | *Car9* siRNA/Br-cAMP | 4 | 0.175 | 0.175 |
| 8D | *Col10a1* mRNA | Control/Control | 4 | 1 | 0.015 |
|  |  | Control/Br-cAMP | 4 | 1.005 | 0.25 |
|  |  | *Car9* siRNA/Control | 4 | 6.512 | 0.236 |
|  |  | *Car9* siRNA/Br-cAMP | 4 | 3.105 | 0.543 |
| 8E | *Epas1* mRNA | Control/Control | 4 | 1 | 0.161 |
|  |  | Control/Br-cAMP | 4 | 2.217 | 0.148 |
|  |  | *Car9* siRNA/Control | 4 | 5.115 | 0.357 |
|  |  | *Car9* siRNA/Br-cAMP | 4 | 9.358 | 2.574 |
