# Supplementary material for: Downregulation of Carbonic Anhydrase IX Promotes Col10a1 Expression in Chondrocytes
Source: PLoS One. 2013 Feb 18;8(2):e56984. doi: 10.1371/journal.pone.0056984 (PMC3575511; doi:10.1371/journal.pone.0056984)
Supplement: Table S6 — Statistical analysis performed for drawing the Figures. (DOC) [file pone.0056984.s006.doc]

**Table S**6. Statistical analysis performed for drawing the Figures.

| Figure | Subject | Comparison | Test | Degree of freedom | Test statistic | *P*-value |  |
| --- | --- | --- | --- | --- | --- | --- | --- |
| 2B | *Col2a1* mRNA | 1 vs 2 | Steel-Dwass test | 15 | T=1.28 | 0.41 |  |
|  |  | 1 vs 3 |  | 15 | T=2.4 | 0.043 |  |
|  |  | 2 vs 3 |  | 15 | T=1.28 | 0.41 |  |
| 2C | *Col10a1* mRNA | 1 vs 2 |  | 15 | T=2.25 | 0.068 |  |
|  |  | 1 vs 3 |  | 15 | T=2.89 | 0.011 |  |
|  |  | 2 vs 3 |  | 15 | T=1.76 | 0.18 |  |
| 2D | *Car9* mRNA | 1 vs 2 |  | 15 | T=2.88 | 0.011 |  |
|  |  | 1 vs 3 |  | 15 | T=2.72 | 0.018 |  |
|  |  | 2 vs 3 |  | 15 | T=0.64 | 0.79 |  |
| 3A | *Car9* mRNA | Control vs *Car9* siRNA | Mann-Whitney *U*-test (Two-tail) | 6 | Z=2.309 | 0.021 |  |
| 3B | Cell proliferation (day 0) | Control vs *Car9* siRNA | Mann-Whitney *U*-test (Two-tail)  (Bonferonni correction) | 35 | Z=1.73 | 0.083 |  |
|  | Cell proliferation (day 2) | Control vs *Car9* siRNA |  | 35 | Z=2.31 | 0.021 |  |
|  | Cell proliferation (day 4) | Control vs *Car9* siRNA |  | 35 | Z=2.31 | 0.021 |  |
|  | Cell proliferation (day 7) | Control vs *Car9* siRNA |  | 35 | Z=1.16 | 0.25 |  |
|  | Cell proliferation (day 10) | Control vs *Car9* siRNA |  | 35 | Z=2.31 | 0.021 |  |

**Table S6. Statistical analysis performed for drawing the Figures (continued).**

| Figure | Subject | Comparison | Test | Degree of freedom | Test statistic | *P*-value |
| --- | --- | --- | --- | --- | --- | --- |
| 3E | *Col2a1* mRNA | Control vs *Car9* siRNA | Mann-Whitney *U*-test (Two-tail) | 6 | Z=1.15 | 0.25 |
| 3F | *Col10a1* mRNA | Control vs *Car9* siRNA |  | 6 | Z=2.31 | 0.021 |
| 3G | *Acan* mRNA | Control vs *Car9* siRNA |  | 6 | Z=2.31 | 0.021 |
| 3H | Alcian Blue | Control vs *Car9* siRNA |  | 6 | Z=2.31 | 0.021 |
| 4A | *Sox5* mRNA | Control vs *Car9* siRNA |  | 6 | Z=0.87 | 0.39 |
| 4B | *Sox6* mRNA | Control vs *Car9* siRNA |  | 6 | Z=0.58 | 0.56 |
| 4C | *Sox9* mRNA | Control vs *Car9* siRNA |  | 6 | Z=1.73 | 0.083 |
| 4D | *Epas1* mRNA | Control vs *Car9* siRNA |  | 6 | Z=2.31 | 0.021 |
| 4G | Intracellular pH | Control vs *Car9* siRNA |  | 6 | Z=0.58 | 0.56 |
| 4H | Extracellular pH | Control vs *Car9* siRNA |  | 6 | Z=0.58 | 0.56 |
| 5A | *Car9* mRNA | Control vs *Car9* siRNA |  | 6 | Z=2.31 | 0.021 |
| 5B | *Col10a1* mRNA | Control vs *Car9* siRNA |  | 6 | Z=2.31 | 0.021 |
| 5C | *Epas1* mRNA | Control vs *Car9* siRNA |  | 6 | Z=2.31 | 0.021 |
| 5D | *Sox5* mRNA | Control vs *Car9* siRNA |  | 6 | Z=2.31 | 0.021 |
| 5E | *Sox6* mRNA | Control vs *Car9* siRNA |  | 6 | Z=2.31 | 0.021 |
| 5F | *Sox9* mRNA | Control vs *Car9* siRNA |  | 6 | Z=2.31 | 0.021 |

**Table S6. Statistical analysis performed for drawing the Figures (continued).**

| Figure | Subject | Comparison | Test | Degree of freedom | Test statistic | *P*-value |
| --- | --- | --- | --- | --- | --- | --- |
| 6B | Cell proliferation (day 0) | Control vs *Car9* OE | Mann-Whitney *U*-test (Two-tail)  (Bonferroni correction) | 35 | Z=0.29 | 0.77 |
|  | Cell proliferation (day 2) | Control vs *Car9* OE |  | 35 | Z=0.29 | 0.77 |
|  | Cell proliferation (day 4) | Control vs *Car9* OE |  | 35 | Z=0.87 | 0.39 |
|  | Cell proliferation (day 7) | Control vs *Car9* OE |  | 35 | Z=1.73 | 0.083 |
|  | Cell proliferation (day 10) | Control vs *Car9* OE |  | 35 | Z=1.44 | 0.15 |
| 6C | *Col2a1* mRNA | Control vs *Car9* OE | Mann-Whitney *U*-test (Two-tail) | 6 | Z=2.31 | 0.021 |
| 6D | *Acan* mRNA | Control vs *Car9* OE |  | 6 | Z=2.31 | 0.021 |
| 6E | *Col10a1* mRNA | Control vs *Car9* OE |  | 6 | Z=0 | 1 |
| 6F | *Epas1* mRNA | Control vs *Car9* OE |  | 6 | Z=0.29 | 0.77 |
| 7A | *Car9* mRNA | Control/Control vs si*Car9*/Control | Steel-Dwass test | 16 | T=2.61 | 0.045 |
|  |  | Control/Control vs Control/si*Epsa1* |  | 16 | T=2.61 | 0.045 |
|  |  | Control/Control vs si*Car9*/si*Epas1* |  | 16 | T=2.61 | 0.045 |
|  |  | si*Car9*/Control vs Control/si*Epsa1* |  | 16 | T=1.15 | 0.66 |
|  |  | si*Car9*/Control vs si*Car9*/si*Epas1* |  | 16 | T=2.61 | 0.045 |
|  |  | Control/si*Epsa1* vs si*Car9*/si*Epas1* |  | 16 | T=2.61 | 0.045 |

**Table S6. Statistical analysis performed for drawing the Figures (continued).**

| Figure | Subject | Comparison | Test | Degree of freedom | Test statistic | *P*-value |
| --- | --- | --- | --- | --- | --- | --- |
| 7B | *Epas1* mRNA | Control/Control vs si*Car9*/Control | Steel-Dwass test | 16 | T=2.61 | 0.045 |
|  |  | Control/Control vs Control/si*Epsa1* |  | 16 | T=2.40 | 0.077 |
|  |  | Control/Control vs si*Car9*/si*Epas1* |  | 16 | T=2.61 | 0.045 |
|  |  | si*Car9*/Control vs Control/si*Epsa1* |  | 16 | T=2.61 | 0.045 |
|  |  | si*Car9*/Control vs si*Car9*/si*Epas1* |  | 16 | T=0.94 | 0.78 |
|  |  | Control/si*Epsa1* vs si*Car9*/si*Epas1* |  | 16 | T=2.61 | 0.045 |
| 7C | *Col10a1* mRNA | Control/Control vs si*Car9*/Control |  | 16 | T=2.61 | 0.045 |
|  |  | Control/Control vs Control/si*Epsa1* |  | 16 | T=2.61 | 0.045 |
|  |  | Control/Control vs si*Car9*/si*Epas1* |  | 16 | T=2.61 | 0.045 |
|  |  | si*Car9*/Control vs Control/si*Epsa1* |  | 16 | T=2.61 | 0.045 |
|  |  | si*Car9*/Control vs si*Car9*/si*Epas1* |  | 16 | T=2.61 | 0.045 |
|  |  | Control/si*Epsa1* vs si*Car9*/si*Epas1* |  | 16 | T=2.61 | 0.045 |

**Table S6. Statistical analysis performed for drawing the Figures (continued).**

| Figure | Subject | Comparison | Test | Degree of freedom | Test statistic | *P*-value |
| --- | --- | --- | --- | --- | --- | --- |
| 8B | cAMP | Control vs *Car9* siRNA | Mann-Whitney *U*-test (Two-tail) | 10 | Z=2.24 | 0.025 |
| 8C | PKA | Control vs *Car9* siRNA |  | 24 | Z=1.04 | 0.297 |
| 8D | *Car9* mRNA | Control/Control vs Control/Br-cAMP |  | 6 | Z=1.44 | 0.15 |
|  |  | *Car9* siRNA/Control  vs *Car9* siRNA/Br-cAMP |  | 6 | Z=2.02 | 0.043 |
| 8E | *Col10a1* mRNA | Control/Control vs Control/Br-cAMP |  | 6 | Z=0 | 1 |
|  |  | *Car9* siRNA/Control  vs *Car9* siRNA/Br-cAMP |  | 6 | Z=2.31 | 0.021 |
| 8F | *Epas1* mRNA | Control/Control vs Control/Br-cAMP |  | 6 | Z=2.31 | 0.021 |
|  |  | *Car9* siRNA/Control  vs *Car9* siRNA/Br-cAMP |  | 6 | Z=2.31 | 0.021 |
